# New Drugs, Appliances, and Things Medical

**Published:** 1893-03-18

**Authors:** 


### NEW DRUGS, APPLIANCES, AND THINGS MEDICAL.

[All preparations, appliances, novelties, etc., of which a notice is desired, should be sent for the Editor, to care of The Manager, 428, Strand, London, W.C.]

### "CHRISTIA TISSUE."

(Thos. Christy and Co., 21, Lime Street, London, E.C.)

This tissue is offered as a "perfect substitute for oiled silk," and similar materials. The great requisites for such a material are impermeability and the power of resisting the action of heat. We find that "Christia" fulfils both of these conditions. It is a smooth, supple, soft material, quite impermeable, not affected by boiling water, nor by alcohol, ether, chloroform, or carbolic acid. It is admirably adapted for medical and surgical uses.

